# Cerebellum, Basal Ganglia, and Cortex Mediate Performance of an Aerial Pursuit Task

**DOI:** 10.3389/fnhum.2020.00029

**Published:** 2020-02-14

**Authors:** Robert J. Gougelet, Cengiz Terzibas, Daniel E. Callan

**Affiliations:** ^1^Department of Cognitive Science, University of California, San Diego, La Jolla, CA, United States; ^2^Swartz Center for Computational Neuroscience, University of California, San Diego, La Jolla, CA, United States; ^3^Multisensory Cognition and Computation Laboratory, Universal Communication Research Institute, National Institute of Information and Communications Technology, Kyoto, Japan; ^4^Center for Information and Neural Networks, National Institute of Information and Communications Technology, Osaka University, Osaka, Japan

**Keywords:** neuroergonomics, fMRI, aviation, flying, affordance competition hypothesis, somatic marker hypothesis, universal cerebellar computation, dysmetria of thought

## Abstract

The affordance competition hypothesis is an ethologically inspired theory from cognitive neuroscience that provides an integrative neural account of continuous, real-time behavior, and will likely become increasingly relevant to the growing field of neuroergonomics. In the spirit of neuroergonomics in aviation, we designed a three-dimensional, first-person, continuous, and real-time fMRI task during which human subjects maneuvered a simulated airplane in pursuit of a target airplane along constantly changing headings. We introduce a pseudo-event-related, parametric fMRI analysis approach to begin testing the affordance competition hypothesis in neuroergonomic contexts, and attempt to identify regions of the brain that exhibit a linear metabolic relationship with the continuous variables of task performance and distance-from-target. In line with the affordance competition hypothesis, our results implicate the cooperation of the cerebellum, basal ganglia, and cortex in such a task, with greater involvement of the basal ganglia during good performance, and greater involvement of cortex and cerebellum during poor performance and when distance-from-target closes. We briefly review the somatic marker and dysmetria of thought hypotheses, in addition to the affordance competition hypothesis, to speculate on the intricacies of the cooperation of these brain regions in a task such as ours. In doing so, we demonstrate how the affordance competition hypothesis and other cognitive neuroscience theories are ready for testing in continuous, real-time tasks such as ours, and in other neuroergonomic settings more generally.

## Introduction

For catching dinner, a mate, or a baseball, the visual tracking and interception of moving targets is a pertinent task to many creatures. Brain-imaging studies that examine this task often operationalize it in an overly simplistic and reductionist manner, using only basic shapes and simple movement, which are far removed from real world experience. The nascent field of neuroergonomics attempts to address this oversimplification by bringing neuroscience into everyday life and the workplace (Parasuraman, 2003). Moreover, recent invocations of ethology in cognitive neuroscience have emphasized consideration of naturalistic real-time behavior and suggest new interpretations of neural data in ethological and ecological contexts (Cisek, 2007; Cisek and Kalaska, 2010), like those of interest in neuroergonomics. In this experiment, we adopt a neuroergonomic and ethological framework and use a robust real-world aviation task with continuous, real-time interactivity to identify brain regions underlying the visual tracking and interception of a moving object.

Previous research on visual tracking and interception of moving targets have identified numerous brain regions involved in visual perception, motor control, prediction, planning, and execution. These brain regions include middle temporal/V5, lateral occipital cortex, inferior parietal lobule, superior parietal lobule, frontal eye field, sensorimotor cortex, supplementary motor area, cerebellum, and basal ganglia (Field and Wann, 2005; Nagel et al., 2006; Ohlendorf et al., 2007; Lencer and Trillenberg, 2008; Senot et al., 2008; Fautrelle et al., 2011; Lungu et al., 2016). While identifying the metabolic activity of specific brain regions in the performance of our task is important, the “affordance competition hypothesis” offers an appealing systems-level and integrative account of how the brain might perform continuous and real-time actions in tasks such as ours and in the world. We believe the affordance competition hypothesis will become increasingly relevant in neuroergonomic contexts for this reason.

The affordance competition hypothesis emphasizes a pragmatic and parallelized role of the brain in the performance of real-time and interactive behavior (for a review, see Cisek, 2007; Cisek and Kalaska, 2010). The affordance competition hypothesis posits that ongoing action selection, i.e. “what to do,” and action specification, i.e. “how to do it,” occurs in a highly distributed and simultaneous manner, with the cerebellum, basal ganglia, and cortex specifically implicated as important network nodes. More specifically, peaks of tuned neural activity pertinent to multiple potential actions in visual, parietal, and premotor cortex are competitively biased by recurrent connections with basal ganglia and prefrontal cortical regions that collect information for action selection, cerebellar attunement, and execution. The integrative and cooperative function of the cerebellum, basal ganglia, and cortex seems evident elsewhere in the literature (Caligiore et al., 2017; Bostan and Strick, 2018).

Concurrent to new interpretations of neural data, the affordance competition hypothesis emphasizes continuous, real-world, real-time, and interactive tasks, in consideration of the evolution of the brain and its naturally time-pressured, complex, and risky ethological circumstances. Recent theory on grounded and embodied cognition echo these sentiments (Wilson, 2002; Barsalou, 2008). It stands to reason that simultaneous action selection and specification, especially among many multiple potential actions, has ethological advantages over the serial processing of complete internal representations and abstractions of the world, as is suggested by contrasting prominent information processing accounts of cognition.

**FIGURE 1:**
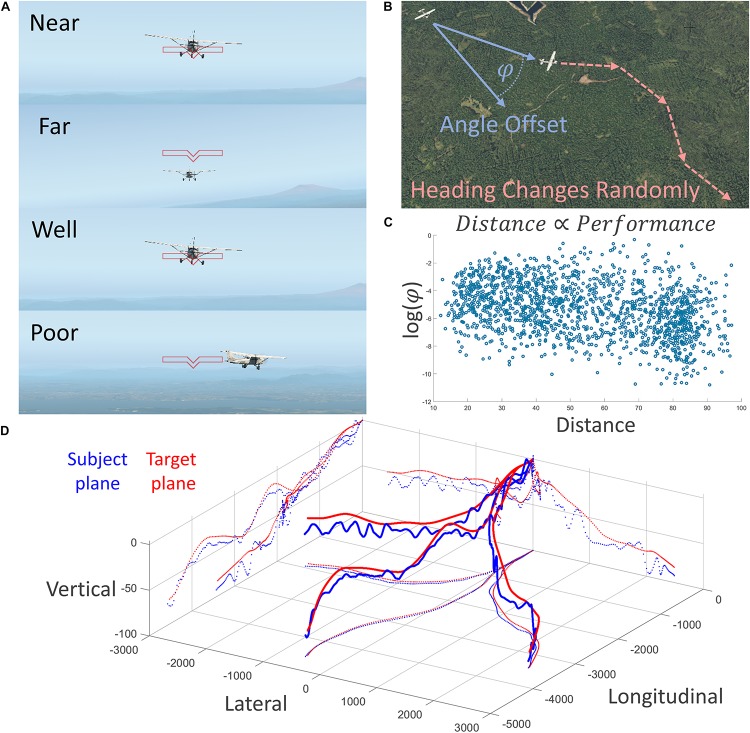
Description of the experimental task conducted in the fMRI scanner. **(A)** First-person depiction of two behavioral variables of interest: experimentally manipulated distance (top two) and performance (bottom two). **(B)** Subjects were tasked with training a crosshair on the target airplane, which changed headings randomly. Performance was measured as log of three-dimensional angle offset; negative is better. **(C)** Behavioral results showed a strong relationship between distance and performance; flying farther away was easier. **(D)** Three-dimensional depiction of three runs of subject and target plane trajectories; note the constant course correction of the subject.

With these ethological considerations of the affordance competition hypothesis in mind, and in an attempt to apply it in a neuroergonomic setting, we designed a three-dimensional and first-person fMRI task during which human subjects maneuvered a simulated airplane in pursuit of a target airplane along constantly changing headings; see Figure 1 for details. This task was originally inspired to be a three-dimensional and more realistic version of the traditional smooth pursuit (Krauzlis, 2004) and shape-tracing tasks (Gowen and Miall, 2007). This task might be imagined as a formation-flying task, wherein the subjects are “following the leader” or it might also be imagined as a non-violent version of a simulated aerial “dog-fight.”

We quantified subject performance of this task as how well the subjects could keep the target airplane in the center of their visual field, measured by the logarithm of the angle offset (in radians) between the three-dimensional vector projected outward from the center of their visual field and the vector projected from the three-dimensional location of the subject airplane to the target airplane (Figures 1A,B). This task was made particularly challenging by imposing smooth but unpredictable heading changes of the leading airplane, as well as imposing smooth changes in both airplanes’ velocities to vary the distance between the two airplanes in an oscillating pattern. See Figure 1D for example traces of the location of both airplanes during three runs of the task.

Under the affordance competition hypothesis, we predict this task, and others like it, will exhibit highly distributed brain activity, with the cerebellum, basal ganglia, and cortex as important network nodes, and as continuous action selection and specification co-occur. An open question under the affordance competition hypothesis is the extent to which ongoing performance and time pressures of the task influence network activity. We compared brain activity when subjects were performing poorly on the task vs. when they were performing well. We also manipulated the ongoing distances between the airplanes and predict that greater time pressure, i.e. closer proximity, will elicit greater network activity as action selection and specification are forced to occur with greater simultaneity. Since both task performance and distance-from-target are continuous variables with no discernible events to time-lock to, we introduce a pseudo-event-related, parametric fMRI analysis approach to test these predictions. Lastly, we briefly review the somatic marker and dysmetria of thought hypotheses, in addition to the affordance competition hypothesis, to speculate on the intricacies of the cooperation of these brain regions in a task such as ours.

## Methods

### Subjects

A total of 24 Japanese subjects from Osaka University and neighboring areas had an average age of 24.4 (*SE* = 1.35) years, five of which were female and all were right-handed. Fourteen of the subjects identified themselves as pilots and were recruited from nearby glider clubs. The other subjects reported experience with first-person video games, e.g. driving games. All subjects had normal or corrected visual acuity using MRI-compatible eye-glasses. All subjects had previously trained extensively on the experimental task, having completed the same task as part of a larger study prior to entering the scanner.

### Procedures

#### The Flying Task

The flying task was designed in X-Plane 9, a versatile and programmable flight simulation software. Subjects flew behind a target plane at experimentally manipulated headings, speeds, and distances, and were tasked to maintain pursuit of the target plane and train a crosshair on the target plane by maneuvering their own plane (Figure 1A). Subjects controlled a fiber-optic flight stick with only pitch and roll axes enabled. Subject and target plane throttles were experimentally manipulated to vary the distance between them. The heads-up display for the subjects was limited to a simple crosshair. Each flying block was 90 s long, followed by a 60 s passive period. Subjects flew four flying blocks. See the following articles for further information regarding the fMRI and MEG compatible flight simulation system utilizing X-Plane (Callan et al., 2012, 2013, 2016a,b; Durantin et al., 2017). Subjects also performed an auditory task simultaneous to the flying task, results from which showed no statistical significance and are not presented here.

#### In the Scanner

Audio and video stimuli for the flying task were presented to the subject within the MRI scanner. Video was projected to a mirror behind the head coil that could be viewed by a mirror mounted on the head coil. MR-compatible headphones were used to present audio (Hitachi Advanced Systems’ ceramic transducer headphones; frequency range 30 – 40,000 Hz; approximately 20 dB SPL passive attenuation). The engine, propeller, and wind sounds of the airplane were constantly playing in the background. This background sound was presented at approximately 85 dBA with the greatest power at 120 Hz with some reduced power at 100 Hz, 155 Hz, 206 Hz, 236 Hz, 275Hz, 310 Hz, 350 Hz, and 466 Hz (recorded using Bruel and Kjaer sound level meter type 2250-S).

#### Scanner Noise

The maximum sound pressure level recorded inside the bore for the multiband EPI sequence used in this study was 95 dBA with a dominant peak at 700 Hz and a lesser one at 2200 Hz (recorded using a microphone on Opto Acoustics MRI compatible noise canceling headphones). The Hitachi Advanced Systems’ headphones used in this study provide approximately 20 dB of passive attenuation. This places the scanner noise at approximately 75 dBA, about 10 dBA lower than the level at which the background noise was presented.

### fMRI

#### Scanning

This fMRI experiment was conducted at the Center for Information and Neural Networks using a Siemens Trio 3T scanner using similar procedures as those reported in Durantin et al. (2017). We used a multiband (factor = 2) gradient-echo EPI sequence employing the blipped CAIPI algorithm (Setsompop et al., 2012). The scanning parameters were the following: Coil = 32 Channel head coil; FOV = 192 mm × 192 mm; Matrix 64 × 64; TR = 1700 ms; TE = 30 ms; FA = 70 degrees; Slice thickness = 3.0 mm no gap (3 mm × 3 mm × 3 mm voxel resolution across the entire brain); Number of slices = 50; Series = Interleaved). Given the low multiband factor of 2 (Preibisch et al., 2015; Todd et al., 2016) combined with the 8 mm smoothing preprocessing step it is unlikely that our multiband scanning procedures adversely affect our results. An entire experimental session consisted of one fMRI session of around 11.5 min (approximately 400 scans). Some of the subjects had a resting state 8-min session (283 scans) and/or a T1 anatomical MRI scan with 1 mm × 1 mm × 1 mm voxel resolution before and/or after the experimental session. The resting state scans were not used in the fMRI analysis. Dummy scans were automatically collected by the Siemens Trio 3T Scanner. Fieldmaps were not collected.

The subjects were instructed to keep their body as still as possible to reduce the degree of head and body movement artifacts. The use of a strap on the forehead and cushions around the head were also used to immobilize the head. The joystick was placed next to the subject in a manner such that minimal movement of the hand and wrist was required to control the continuous movement of the airplane, in order to reduce potential body movement related artifacts.

### Preprocessing

The fMRI scans were preprocessed using functions within SPM8 (Wellcome Department of Cognitive Neurology, UCL). Images from the experimental session were realigned, unwarped, and spatially normalized to a standard space using a template EPI image (2 mm × 2 mm × 2 mm voxels) provided in SPM, and were smoothed using an 8 mm × 8 mm × 8 mm FWHM Gaussian kernel. Spatial normalization was conducted by using the mean EPI image (after realignment and unwarping preprocessing steps) as the source image, and the SPM MNI EPI image (given with the SPM software) as the template image to normalize to. One advantage of using the mean EPI image rather than an anatomical T1 or T2 image for normalization is that it avoids the necessary extra step of having to co-register the T1 (or T2) image to the same space as the acquired EPI images (which could result in some degree of error) in order to apply the normalization parameters to the entire set of EPI used for further SPM analysis. No subjects were excluded because of excessive head motion. All subjects had less than 3 mm translation and 5 degrees rotation deviations between scans. The realignment parameters were used as regressors of non-interest to account for small deviations in head movement across scans. Auto-regression was used to correct for serial correlations. High pass filtering (cutoff period 240 s; twice the maximum duration between identical condition stimuli) was carried out to reduce the effects of extraneous variables (scanner drift, low frequency noise, etc.).

### Analysis

Regional brain activity for each subject for the various conditions was assessed using a general linear model (GLM) employing a pseudo-event-related design. Given the continuous nature of the two variables of interest, flying performance and flying distance, there were no overt experimental events to time-lock the analysis to. Meanwhile, a block design would not provide much temporal precision below the 90 s flying blocks. Instead, randomly time-jittered pseudo-events were generated throughout the time course of the task. To prevent temporal dependence effects, the interval between pseudo-events was randomly generated from an exponential distribution with a minimum of 3 s and a maximum of 15 s. In order to investigate a linear relationship between the time-varying values of the two variables of interest and brain activity, a parametric modulator approach was employed (for an example, see Nagel et al., 2006).

Parametric modulation is implemented as amplitude weighting of the impulse functions corresponding to pseudo-event onset by the values of the continuous “parametric modulators” performance and distance. Flying performance took continuous values between 1 and 2 radians corresponding to the angle offset between the center of the visual field and the location of the center of the target airplane. Flying distance took continuous values between approximately 10 and 150 meters. These values were intrinsically normalized relative to each subject’s average performance and distance. The canonical hemodynamic response function (HRF) was convolved with the parametrically modulated pseudo-event onset impulse functions and represented in the GLM to account for lag in the Blood Oxygenation Level Dependent (BOLD) response.

Two experimental regressors (flying performance and distance) were included in the GLM as parametric modulators. Including both parametric modulators in the same GLM allows for the contribution for each parameter to be determined while removing the effect of the other. This is because the parameter that is not being used for the contrast under investigation will be treated as a regressor of non-interest and its variance will be removed from the signal (Kiebel and Holmes, 2003). For two contrasts, the parametric modulators were made negative to identify metabolic activity that exhibits the opposite relationship with the parametric modulators, i.e. identify regions that are more active when performance worsens or when distance decreases. Six head-realignment parameters were also included in the GLM for all analyses as regressors of non-interest to account for artifacts in head movement correlations. An additional regressor corresponding to an unrelated auditory task was used to regress out potential effects of this task (see Supplementary Material). Fixed effects analyses were conducted for each subject. Random effects analyses were conducted across subjects for the contrasts of interest given below using t-tests within SPM8.

## Results

### Behavior

Flying performance was quantified as a three-dimensional angle offset, in radians, and was transformed logarithmically in preparation for a parametric statistical test, and showed a statistical correlation with experimentally manipulated distance; *r*(1526) = −0.33, *p* = 7.39 × 10^–41^ (Figure 1C). Subjects performed better when farther away from the target. This relationship survives with similarly strong results after detrending to remove time-series dependence effects *r*(1525) = −0.31, *p* = 2.53 × 10^–36^.

### fMRI

**FIGURE 2:**
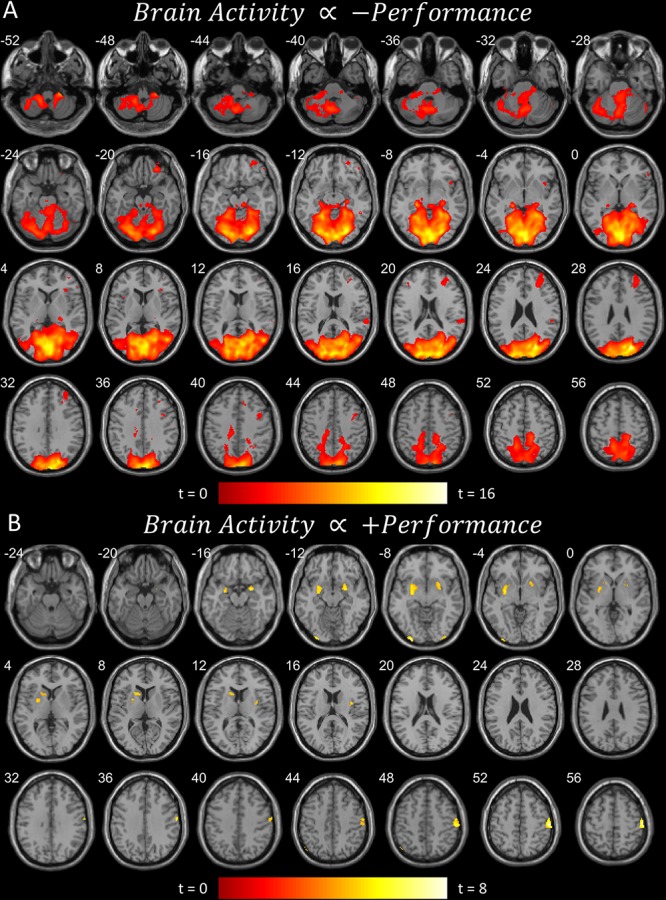
**(A)** Brain areas that become more active when performance worsens, or less active when performance improves (pFDR < 0.05 peak level corrected across entire brain) rendered on anatomical MRI slices using xjView toolbox (https://www.alivelearn.net/xjview). Note cerebellar and cortical activity. **(B)** Brain areas that become more active when performance improves, or less active when performance worsens. Same rendering as **(A)**. Note basal ganglia activity.

**FIGURE 3:**
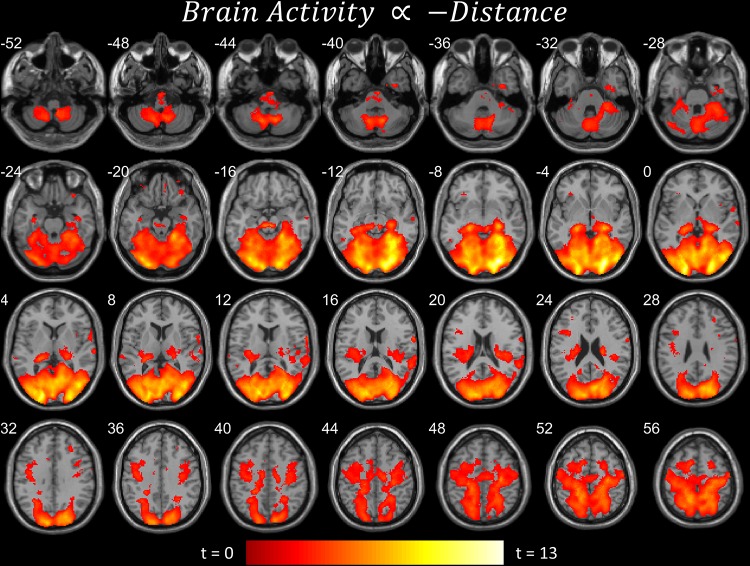
Brain areas that become more active when near the target. Significant parametric modulation (pFDR < 0.05 peak level corrected across entire brain) rendered on anatomical MRI slices using xjView toolbox (https://www.alivelearn.net/xjview). Note widespread, distributed activity, particularly in cerebellum and cortex.

As can be seen in Figure 1C, there is a small (*r* = −0.33) but significant correlation between performance and distance. By including these two parametric modulators in the same GLM it is possible to treat the variance of one parametric modulator as an effect of non-interest and remove it from the signal to better determine the unique contribution of the other parametric modulator for each contrast of interest. The results reported in Table 1 and Figures 2, 3 report contributions for each parametric modulator and contrast of interest.

**TABLE 1:** MNI coordinates for selected cluster maxima (>8 mm apart) of brain activity in presented figures.

Figure	Neurosynth keywords (Yarkoni et al., 2011)	MNI			*T*-value	# of Voxels
		*x*	*y*	*z*		
**Figure 2A – Performance**						
	Vision, occipital cortex, motion	22	−82	28	15.72	42797
	Occipital, visual, fusiform	26	−78	−12	12.39	
	Early visual, v1, lingual gyrus	4	−90	−2	12.10	
	Orbitofrontal cortex, cognitive control	32	38	−22	5.26	184
	Orbitofrontal cortex	30	54	−16	3.65	
	Dorsolateral prefrontal, tasks	30	40	26	4.19	460
	Noxious, prefrontal	34	54	22	2.98	
	Dorsolateral prefrontal, memory tasks	32	46	16	2.88	
	Shapes, shifting, frontostrial, occipital temporal	52	−60	−28	4.11	48
	Fusiform face, cortex cerebellum	50	−50	−28	3.08	
	Cerebellar, passive viewing, finger movement	50	−68	−26	3.02	
	Premotor, motor	64	−30	18	3.90	121
	Somatosensory, secondary somatosensory, pain, tactile	54	−30	24	2.87	
	Temporoparietal junction, tasks, middle frontal gyrus	40	8	40	3.17	112
	Frontal eye field, action, eye movements	38	0	44	3.07	
	Anterior insula, temporal difference learning, pain	34	30	8	3.13	36
	Motor cortex, primary motor, hand	−32	−26	70	2.91	54
	Insula, pain, response inhibition	40	14	−6	2.90	37
**Figure 2B + Performance**						
	Finger somatosensory cortex	58	−22	52	7.97	393
	Hand somatosensory cortex	54	−26	60	6.99	
	Primary somatosensory cortex	56	−14	52	6.05	
	Visual motion	−30	−100	−8	7.26	66
	Amygdala, fear	24	0	−14	6.07	212
	Putamen, losses, striatum, reward	26	8	−10	4.87	
	Putamen, motor, basal ganglia	26	6	0	4.21	
	Amygdala, fear, putamen	−26	−6	−8	5.62	346
	Striatum, putamen, reward	−28	4	−6	5.49	
	Putamen, basal ganglia, motor	−26	0	4	5.03	
	Caudate, dorsal striatum	−12	14	10	5.37	87
	Striatum, putamen, ventral striatum	−20	12	−2	4.15	
	Insular cortex, noxious, nociception	36	−6	14	4.96	41
**Figure 3 – Distance**						
	Occipital, visual, ventral visual, object	26	−82	−8	12.62	70194
	Reading, visually presented, orthographic	22	−94	−4	11.09	
	Early visual, visual, word form	−22	−98	0	10.78	
	Visual cortex, v1	−20	−96	4	10.38	
	Fusiform, navigation, objects	32	−50	−10	9.87	
	Fusiform, objects, parahippocampal	28	−52	−10	9.29	
	Visual, objects, fusiform	46	−78	−2	9.27	
	Disgust, occipitotemporal, agent	−10	−94	−12	9.07	
	Serotonin, negative emotion, noxious, dorsolateral	28	34	32	3.36	71
	Temporal pole, mentalizing, neutral	−34	−14	−24	3.32	55
	Auditory cortex, superior temporal	−66	−26	8	2.92	43
	Listening, middle cortex, auditory	66	−12	−12	2.86	30

The first contrast (Figure 2A) shows the parametric modulation of brain activity by poor task performance. Large areas of the frontal and visual cortices and cerebellum were activated. The second contrast (Figure 2B) shows the parametric modulation of brain activity relative to good task performance. The basal ganglia are clearly identified, together with visual and insular cortices. The third contrast (Figure 3) shows an *increase* in parametric modulated activity by distance. The results are similar to that of the flying contrasts; except less frontal activity, and greater activity in the parietal and somatosensory cortices. No significant voxels were found showing a *decrease* in parametric modulated activity by distance. A separate event-related analysis design targeting the events of the secondary auditory task produced no significant voxels of activation. Contrasts testing the interaction between the secondary auditory task and both performance and distance of the current model also produced no significant results (see Supplementary Material).

## Discussion

Activity concurrent with poor performance (Figure 2A) on the task was found primarily in cortical and cerebellar regions. Activity concurrent with performing well (Figure 2B) on the task was found primarily in the basal ganglia, as well as in primary visual and motor cortices. As time-pressure increased, and distance-to-target closed, widespread cortical and cerebellar activity, particularly in parietal and somatosensory cortices, increased (Figure 3). The results of our experiment seem to support the affordance competition hypothesis (Cisek, 2007; Cisek and Kalaska, 2010), which claims that the cerebellum, basal ganglia, and cortex are involved in ethological and ecologically relevant continuous, real-time tasks such as ours.

One striking result is the isolated involvement of the basal ganglia when flying well. Encouragingly, Durantin et al. (2017) found similar results to ours for flying well; the basal ganglia, namely the putamen and caudate, were active when their subjects were performing their flying task well. Durantin et al. (2017) also found similar results to ours for poor flying performance; with widespread posterior and cerebellar activity and notable activity in the right prefrontal regions, though orbitofrontal activity is absent in their results. On the other hand, brain activity regarding first-person flying in Callan et al. (2012) showed similar right (and left) prefrontal activity together with orbitofrontal activity and widespread posterior and cerebellar activity. Our results are also very similar to the involvement of the cerebellum, basal ganglia, and cortex in maintaining a safe driving distance (Uchiyama et al., 2003). While our results reproduce previous findings and generally confirm the affordance competition hypothesis, future work is necessary to elucidate the intricacies of the cooperation of these brain regions, but we provide some speculation below.

### Cerebellum and Dysmetria of Thought

The cerebellum is often understood as a clock or “time machine” (Bareš et al., 2019). The cerebellum is comprised of a unique and specialized neuronal architecture that provides a mechanism by which neuronal signals can be temporally manipulated and synchronized (Ivry and Keele, 1989; Palay and Chan-Palay, 2012), offering a neural substrate dedicated to temporal information processing. In particular, the cerebellum appears to encode signal predictability, receiving gated input dependent upon whether such input is expected (Lawrenson et al., 2016), thereby reflecting its important role as a mechanism of signal comparison and feedback control, especially in motor timing (Eccles, 2013).

Indeed, the cerebellum appears highly involved in the process of the perception of time, itself (Ivry and Schlerf, 2008). Moreover, neural disorders such as Parkinson’s and spinocerebellar ataxia can disrupt the cerebellum’s timing functionality, leading to deficits in motor timing (Bareš et al., 2010, 2011). These deficits might also occur at a network level, namely in cervical dystonia, potentially interfering with the cooperation between the cerebellum and basal ganglia (Filip et al., 2017).

Under the “dysmetria of thought” hypothesis, the unique neural architecture of the cerebellum appears to support a non-conscious “harmonizing” among converging neural signals of internal representations, external stimuli, and self-generated responses (Schmahmann, 2010). Such a “universal cerebellar computation” is useful in not just motor, but cognitive and autonomic/emotional contexts as well (Schmahmann, 1996; Buckner, 2013), wherein a dysfunctional cerebellum can lead to broad yet selective pathologies (Schmahmann, 2004). In line with the dysmetria of thought hypothesis, the cerebellum therefore seems perhaps relevant to our task as a hub for the harmonizing of eye and hand motor decision-making, internal and external representations of self, target, and environment, and perhaps motivational priorities and emotional states as well.

### Cortex and Somatic Markers

We observed widespread cortical activity during poor performance and near distance-from-target. The prefrontal cortex is suggested to be responsible for the maintenance of task-relevant information over time (Miller and Cohen, 2001). While inaccessible via the current task and analysis design, we speculate the activity we observed in the dorsolateral prefrontal cortex, as well as in the visual, orbitofrontal, and insular cortices, mediates the organization of task-relevant internal and external representations for input into the activated cerebellum, wherein its inputs are “harmonized” for subsequent output to premotor and motor cortices.

Under the “somatic marker hypothesis,” the ventromedial cortex is a frontal cortical region suggested to use its relationship with the amygdala and insular/somatosensory cortices to guide somatic state-dependent decision making (Bechara et al., 2000). We found significant metabolic activity in these regions. Our first-person task, with lifelike continuous responsiveness and engagement, likely invokes somatic markers that guide action, perhaps indicating how the airplane becomes an “extension” of the body (Callan et al., 2012). Moreover, Menon and Uddin (2010) have proposed an important role for the insular cortex as facilitating access to attentional and working memory resources of large-scale cortical networks, as well. We suspect that the insular cortex activity we found during poor performance is concurrent with the aversive state of being out of control of the airplane, creating an aforementioned somatic marker (Bechara et al., 2000) that instigates widespread cerebellar and cortical network reorganization (Menon and Uddin, 2010).

### Basal Ganglia

The basal ganglia are best known for their involvement in the performance and acquisition of goal-directed behavior and reward processing, involving large networks of functionally parallel cortical and subcortical circuits (Haber, 2003; Haber and Knutson, 2010). The basal ganglia have also been broadly implicated in the inhibition of competing, and disinhibition of goal-directed, motor programs (Mink, 1996). Current suggestions of the mechanism by which the basal ganglia perform these functions is through the selection and inhibition of cortical and subcortical signals via internal reentrant loops across mainly parallel circuits (Lanciego et al., 2012).

From the above, we therefore believe the basal ganglia is in the position to balance motivational and arousal decision factors in the performance of neuroergonomic tasks such as ours. We also speculate that, during improved performance, the basal ganglia might be creating a rewarding somatic marker together with a well-harmonized state of cooperation between visual and motor cortices. At the least, the cooperation between the basal ganglia and cerebellum regarding motor timing and coordination is evident in the literature (Middleton and Strick, 2000), and basal ganglia activity in a fine motor control task such as ours is reasonable.

### Other Findings

Regarding behavior, Figure 1C shows that the distance between the subject plane and target plane is related to performance on the task. Since distance was experimentally manipulated, flying farther away from the target seems to make the task easier. Our explanation is that subjects have less time to respond to directional changes of the target plane when the target plane is nearer to them. We also suggest that the continuously changing distance between the two planes elicits a continuously changing degree of how “offline” or “online” the task is. When the distance between the two planes is large, the subjects revert to a more “offline” and relaxed mental state, whereas when the distance is small the subjects become more engaged and “online.” We think this explains why we found no significant fMRI activity when the subjects were far from the target plane but vast activity when the subjects were near. This is also further evidence for the affordance competition hypothesis, such that distributed network activity and action selection and specification are most prominent when distances are near and the greatest time pressures, as found in ethological contexts, are imposed.

To supplement our results, we obtained NeuroSynth (Yarkoni et al., 2011) keywords associated with the locations of SPM cluster maxima we list in Table 1 to help indicate the potential brain region and/or related cognitive phenomena. Most of the keyword matches are as expected. One oddity is the activation of brain regions often implicated in the processing of noxious stimuli and pain; mainly originating in the insular cortex. This motivates our speculation that such insular activity may act as a somatic marker, and we suggest that the insula might play a more prominent role in neuroergonomic contexts in general.

Another oddity is the orthographic and reading-like phenomenology that the NeuroSynth keywords suggest when the two planes are far apart (Table 1 and Figure 3). The subjects are perhaps scanning and projecting small trajectories upon the airplane, treating it as a distant object with some agency. There also seems to be a shift in activity to auditory regions, perhaps indicating a shift of attention toward the unrelated auditory task that subjects were simultaneously performing, though additional analyses focusing on the auditory task produced no significant results after correction for multiple comparisons.

### Future Directions in Neuroergonomics

Our above results and discussion have implications toward the field of neuroergonomics. We believe the application of the various cognitive neuroscience theories such as affordance competition hypothesis (Cisek and Kalaska, 2010), dysmetria of thought and its universal cerebellar computation (Schmahmann, 2010), and the somatic marker hypothesis (Bechara et al., 2000), could provide a richer understanding of more ethological and ecologically relevant neuroergonomic tasks, such as ours. Future work in aviation, and neuroergonomics more generally, will likely benefit from the testing and application of these theories.

We also believe that the pseudo-event-related parametric fMRI design we suggest here will be an important method for future work in neuroergonomics, which will likely have continuous, real-time tasks with continuously fluctuating environmental or human factors like performance, workload, or fatigue. To encourage the engineering and design component of neuroergonomics and human factors, we hope these results and discussion can be applied toward new trainings, interventions, or interface designs. An objective measure of flying performance would provide a new dimension for distinguishing otherwise equally performing pilots/operators (Kane and Engle, 2002). As suggested elsewhere (Gougelet, 2019), future work might also involve the translation of less tractable to more tractable measures (e.g. fMRI to MEG, MEG to EEG, MEG to fNIRS) for eventual integration into everyday situations; thereby supporting the aims of neuroergonomics as a field of study.

## Data Availability Statement

The datasets generated for this study are available on request to the corresponding author.

## Ethics Statement

This study was carried out in accordance with the recommendations of National Institute of Information and Communications Technology (NICT) Human Subject Review Committee with written informed consent from all subjects. All subjects gave written informed consent in accordance with the Declaration of Helsinki. The protocol was approved by the National Institute of Information and Communications Technology (NICT) Human Subject Review Committee.

## Author Contributions

RG was the primary author of this publication, devising and running the experiment, and performing analysis and writing. DC oversaw and contributed to all aspects that RG contributed to. CT provided the source code and support for experimental design and implementation.

## Conflict of Interest

The authors declare that the research was conducted in the absence of any commercial or financial relationships that could be construed as a potential conflict of interest.

## Supplementary Material

FIGURE S1 Example design matrix.

FIGURE S2 Null result – IX auditory task × negative distance.

FIGURE S3 Null result – IX auditory task × negative performance.

FIGURE S4 Null result – IX auditory task × positive distance.

FIGURE S5 Null result – IX auditory task × positive performance.

FIGURE S6 Significant result – negative distance – Figure 3.

FIGURE S7 Significant result – negative performance – Figure 2A.

FIGURE S8 Null result – positive distance.

FIGURE S9 Significant result – positive performance – Figure 2B.
