# Supplementary figures and images for: Cerebellum, Basal Ganglia, and Cortex Mediate Performance of an Aerial Pursuit Task
Source: Front Hum Neurosci. 2020 Feb 14;14:29. doi: 10.3389/fnhum.2020.00029 (PMC7033450; doi:10.3389/fnhum.2020.00029)

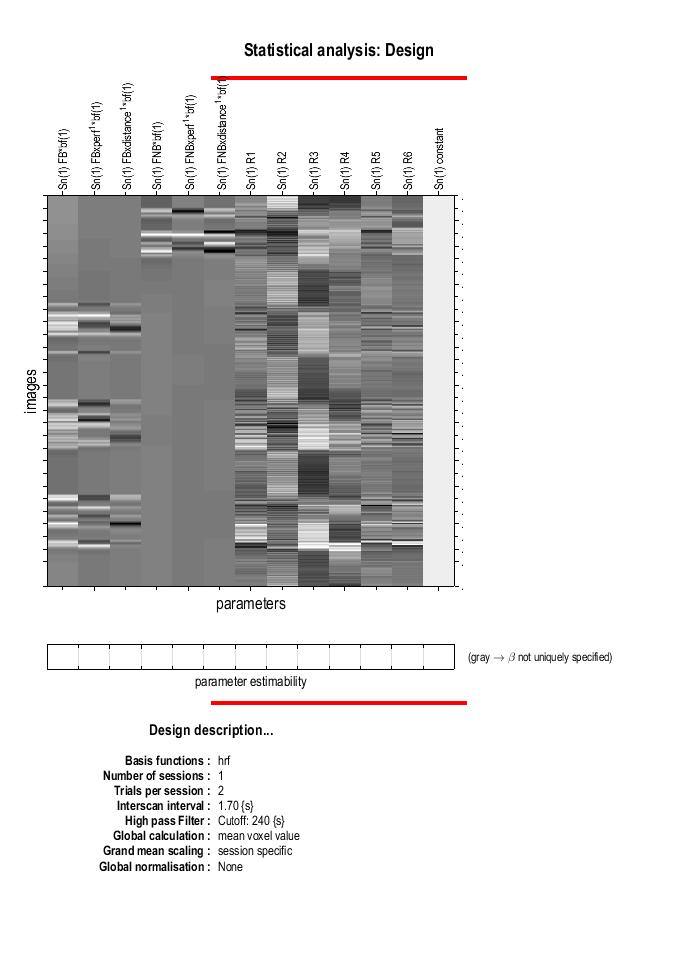

Supplement: FIGURE S1 — Example design matrix. [file Image_1.JPEG]

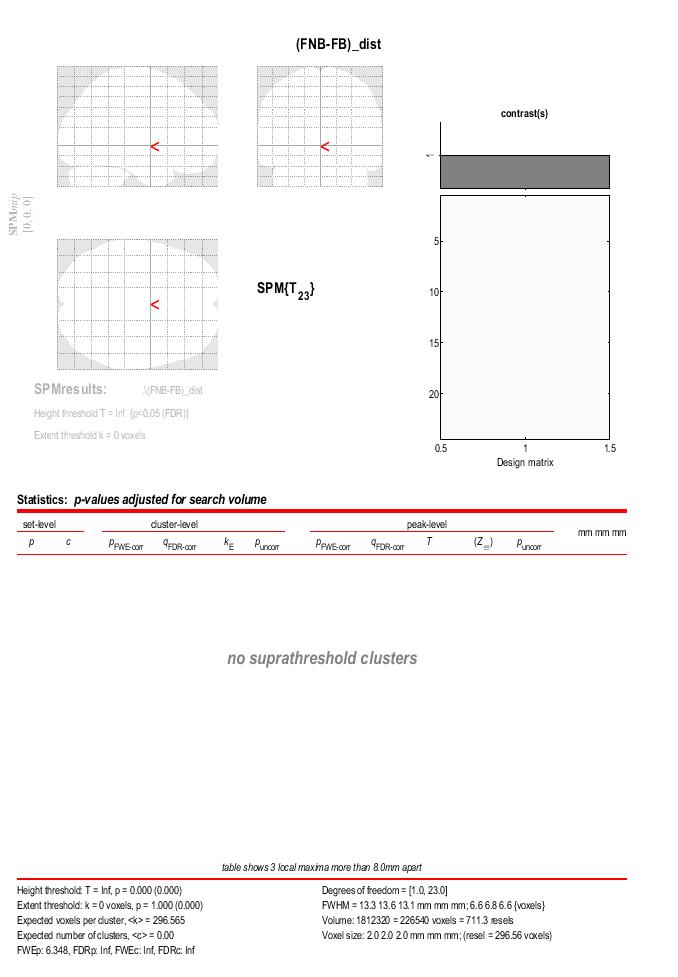

Supplement: FIGURE S2 — Null result – IX auditory task × negative distance. [file Image_2.JPEG]

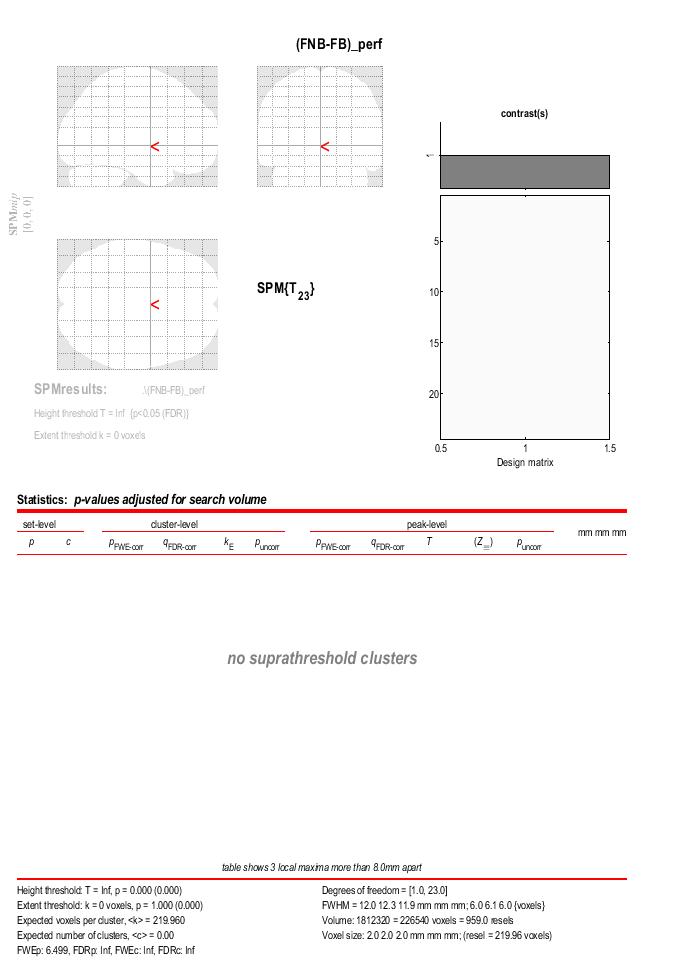

Supplement: FIGURE S3 — Null result – IX auditory task × negative performance. [file Image_3.JPEG]

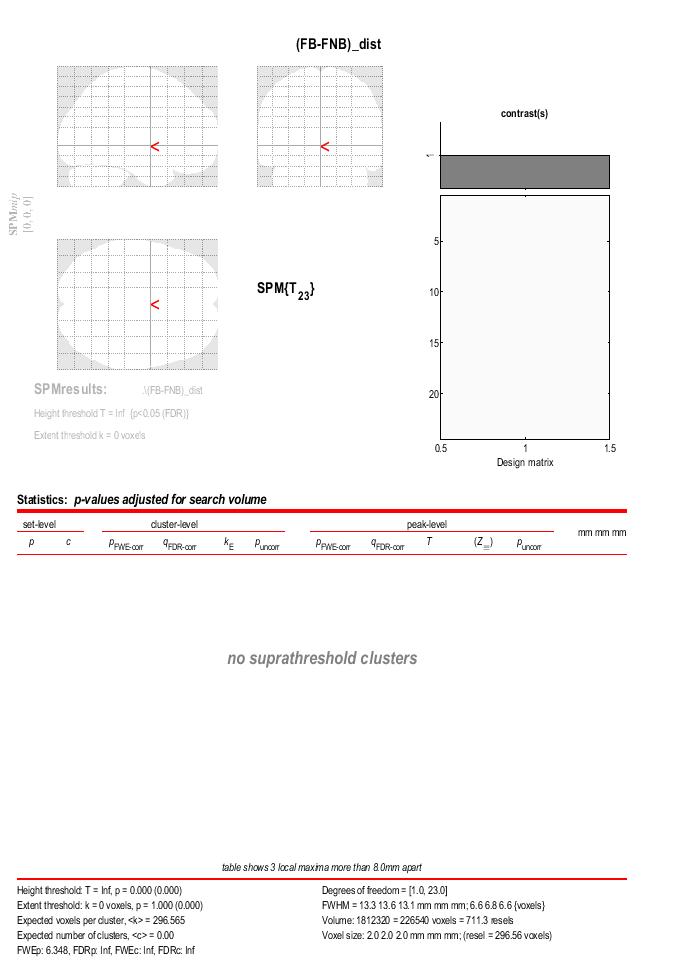

Supplement: FIGURE S4 — Null result – IX auditory task × positive distance. [file Image_4.JPEG]

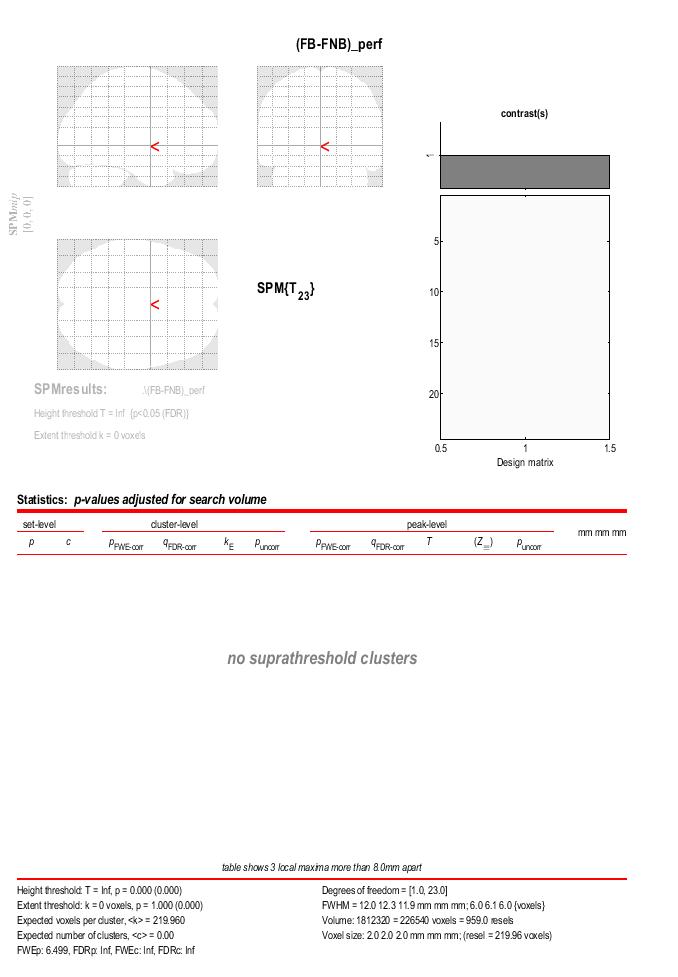

Supplement: FIGURE S5 — Null result – IX auditory task × positive performance. [file Image_5.JPEG]

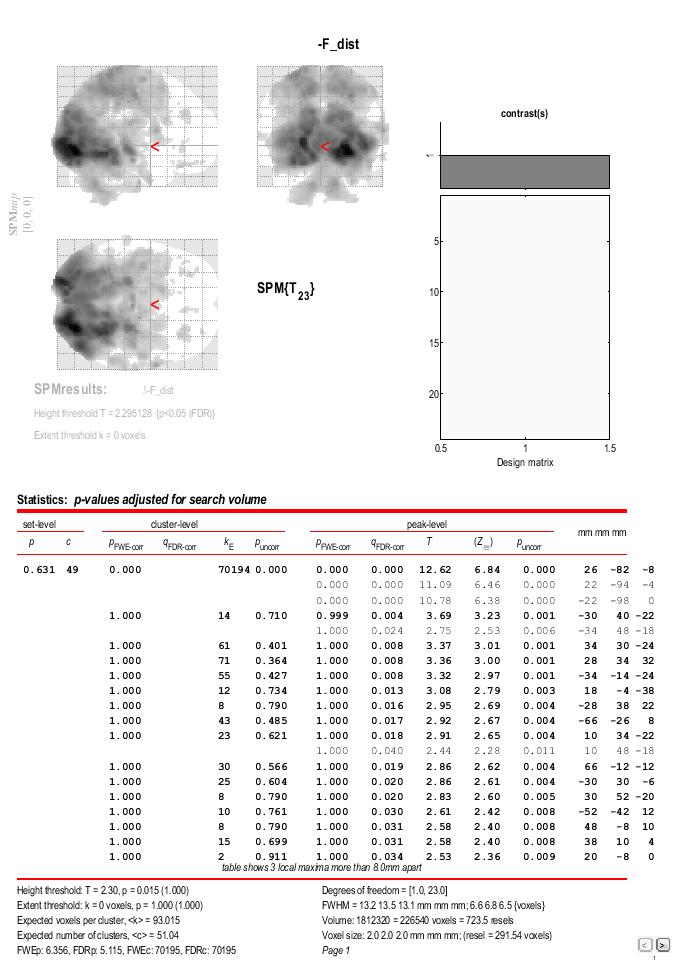

Supplement: FIGURE S6 — Significant result – negative distance – Figure 3. [file Image_6.JPEG]

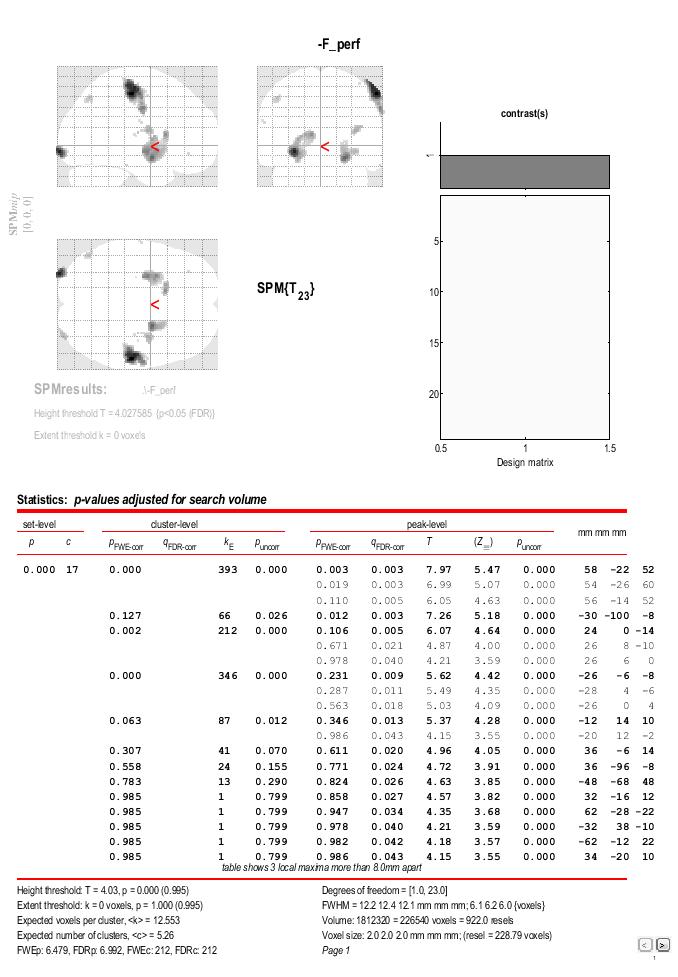

Supplement: FIGURE S7 — Significant result – negative performance – Figure 2A. [file Image_7.JPEG]

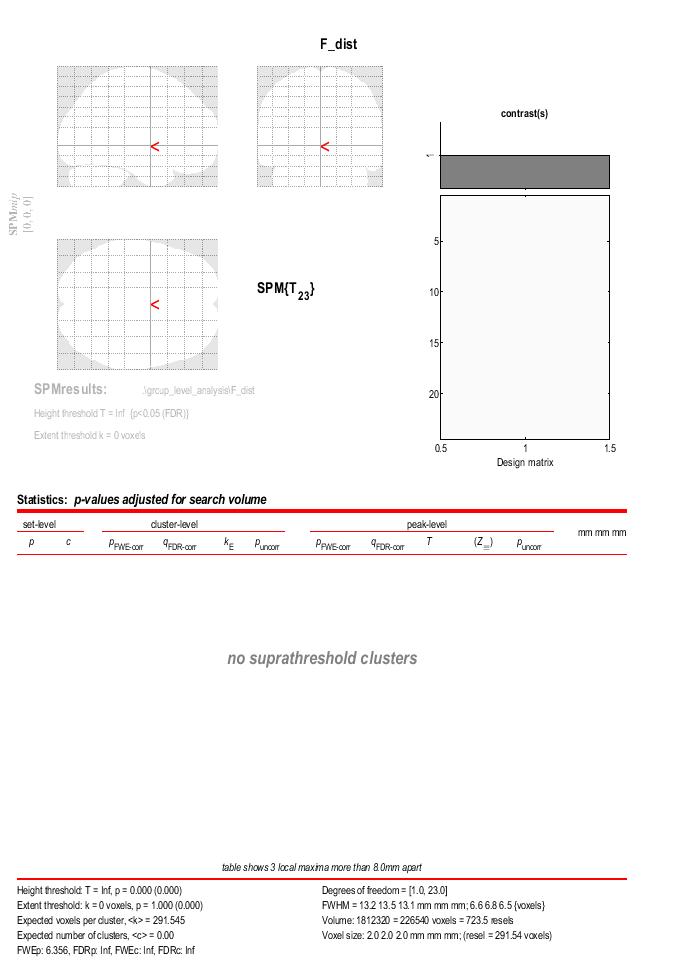

Supplement: FIGURE S8 — Null result – positive distance. [file Image_8.JPEG]

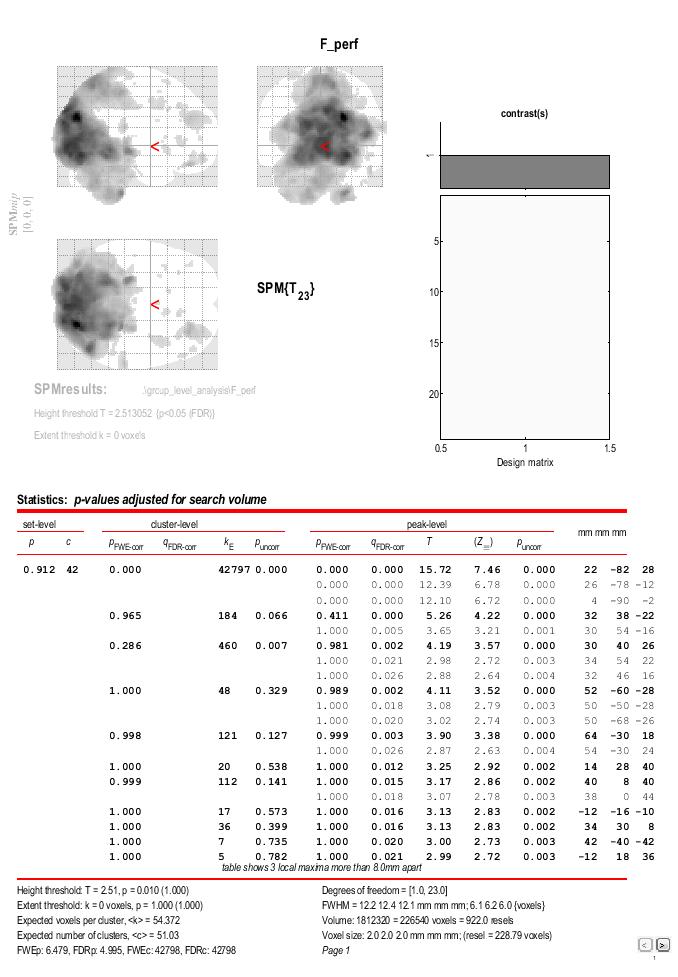

Supplement: FIGURE S9 — Significant result – positive performance – Figure 2B. [file Image_9.JPEG]
